# Not only cardiovascular, but also coordinative exercise increases hippocampal volume in older adults

**DOI:** 10.3389/fnagi.2014.00170

**Published:** 2014-08-04

**Authors:** Claudia Niemann, Ben Godde, Claudia Voelcker-Rehage

**Affiliations:** ^1^Jacobs Center on Lifelong Learning and Institutional Development, Jacobs University BremenBremen, Germany; ^2^AgeAct Research Center, Jacobs University BremenBremen, Germany

**Keywords:** brain aging, cardiovascular fitness, hippocampus, motor fitness, physical activity

## Abstract

Cardiovascular activity has been shown to be positively associated with gray and white matter volume of, amongst others, frontal and temporal brain regions in older adults. This is particularly true for the hippocampus, a brain structure that plays an important role in learning and memory, and whose decline has been related to the development of Alzheimer’s disease. In the current study, we were interested in whether not only cardiovascular activity but also other types of physical activity, i.e., coordination training, were also positively associated with the volume of the hippocampus in older adults. For this purpose we first collected cross-sectional data on “metabolic fitness” (cardiovascular fitness and muscular strength) and “motor fitness” (e.g., balance, movement speed, fine coordination). Second, we performed a 12-month randomized controlled trial. Results revealed that motor fitness but not metabolic fitness was associated with hippocampal volume. After the 12-month intervention period, both, cardiovascular and coordination training led to increases in hippocampal volume. Our findings suggest that a high motor fitness level as well as different types of physical activity were beneficial to diminish age-related hippocampal volume shrinkage or even increase hippocampal volume.

## INTRODUCTION

It has repeatedly been shown that physical activity is positively related to brain structure and function (see Kramer et al., 2006; Hillman et al., 2008; Park and Reuter-Lorenz, 2009) as well as cognitive performance (for a review see Etnier et al., 2006) and that it might reduce the risk of developing dementia (Sumic et al., 2007; Erickson et al., 2012). Particularly the hippocampal formation has been in the focus of these studies investigating the positive effect of physical activity on brain volume because this brain structure is thought to be significantly involved in diseases related to memory impairment like, e.g., Alzheimer’s disease (Driscoll et al., 2003; Barnes et al., 2009). Whereas positive correlations between cardiovascular activity and hippocampal volume have been shown, it is unknown whether this applies to other types of physical activity like, e.g., coordination training, as well. With this paper we aim to extend the knowledge about the effects of different physical activity interventions in older adults and its potential to diminish the decline in hippocampal volume during the aging process.

### THE HIPPOCAMPUS AND ITS SHRINKAGE ACROSS THE ADULT LIFESPAN

The hippocampal formation as part of the limbic system is located in the medial temporal lobe. It is highly involved in processes of episodic memory formation (Tulving and Markowitsch, 1998; Van Petten, 2004) and spatial navigation (O’Keefe, 1990; Maguire et al., 2000). Both cognitive dimensions are especially vulnerable to performance loss in late adulthood (cf. Hedden and Gabrieli, 2004). Recently, the hippocampus has also been associated with motor sequence consolidation (Albouy et al., 2008). Over the adult lifespan, on average, hippocampal volume shrinks about 0.86% per year, but this development is highly non-linear (Raz et al., 2004b). Whereas for adults below the age of 50 annual hippocampal volume reductions of only 0.51% were observed, adults above the age of 50 revealed a much steeper annual volume decline of 1.18% (Raz et al., 2004b). These volume reductions are mainly attributed to reductions in the neuropil part of the brain structure and – to a smaller extend – to cell body shrinkage and changes in vascularization (Thomas et al., 2012).

### EFFECTS OF CARDIOVASCULAR FITNESS/TRAINING ON THE HIPPOCAMPUS

The hippocampus seems to be especially sensitive to cardiovascular activity. This has been demonstrated in research with rodents (e.g., van Praag et al., 1999, 2005; Kronenberg et al., 2006; Pereira et al., 2007; Fabel and Kempermann, 2008; Kempermann et al., 2010) and humans (e.g., Pereira et al., 2007; Erickson et al., 2009, 2010, 2011). Rodent studies revealed two main mechanisms to be responsible for structural benefits after cardiovascular training: the enhancement of capillary density (angiogenesis; Black et al., 1990) and the generation of new neurons (neurogenesis; van Praag et al., 1999), whereby the latter is specifically restricted to the dentate gyrus. Typically, old rodents and humans reveal a substantial decline in hippocampal neurogenesis (approx. 10-fold for rodents, cf. Ben Abdallah et al., 2010; and approx. fourfold for humans, cf. Spalding et al., 2013). In old rats the rate of decline in neurogenesis could be halved by 1 month of cardiovascular training and this reduced rate of decline was associated with better spatial learning abilities (van Praag et al., 2005; Kronenberg et al., 2006). These findings indicate a beneficial influence of cardiovascular activity on hippocampal volume and function even in older ages.

In a human sample aged 59–81 years, larger hippocampal volume mediated the relationship between better cardiovascular fitness and higher spatial memory performance (Erickson et al., 2009); and 12 months of cardiovascular training were associated with an increase in hippocampal volume by about 2% in older adults (Erickson et al., 2011). This increase in hippocampal volume was associated with improvement of cardiovascular fitness (VO_2_ max) and spatial memory performance, respectively (Erickson et al., 2011).

### EFFECTS OF MOTOR FITNESS AND COORDINATION TRAINING ON THE BRAIN

Physical activity, however, does not only comprise exercises that require metabolic and energetic processes, like cardiovascular training, but also motor-demanding exercises that require perceptual and higher-level cognitive information processing. This is, for example, true for coordinative exercises, like balance, eye–hand coordination or leg–arm coordination tasks (cf. Voelcker-Rehage and Niemann, 2013). Research on animals suggests that effects of motor-demanding activity on brain metabolism differ from those of cardiovascular activity (Black et al., 1990; Anderson et al., 1994). A motor-demanding training based on coordinative skills (e.g., crossing obstacles, balancing on ropes, etc.) facilitated synaptic growth and re-construction (synaptogenesis; Black et al., 1990; Seo et al., 2010). It also led to a higher degree of survival of newly generated cells than cardiovascular exercise (cf. above Curlik et al., 2013). Results from studies with older adults point in a similar direction. They revealed that motor fitness (e.g., action speed, reaction speed, balance) and coordination training facilitate brain function (particularly in those areas related to visual–spatial processing) and cognitive performance in older adults (Voelcker-Rehage et al., 2010, 2011). The associations of motor fitness with and the influences of coordination training on brain volume and specifically on hippocampal volume in older adults have not been investigated so far.

### RESEARCH QUESTIONS AND HYPOTHESES

In a first step, analyzing a cross-sectional sample of older adults, we analyzed the relationship between metabolic/cardiovascular fitness and the volume of the hippocampus (Erickson et al., 2009). Moreover, for the first time, we tested the hypothesis that motor fitness is also associated with hippocampal volume in older adults. Based on our own findings revealing a positive association of motor fitness and brain function, we assumed to find a positive relationship between motor fitness and hippocampal volume as well. This seems particularly reasonable as the hippocampus is involved in spatial navigation and motor sequence consolidation (O’Keefe, 1990; Albouy et al., 2008), two processes probably associated with motor fitness.

In the second step, we analyzed interventional data from a subsample of the same dataset to investigate the effects of a 12-month cardiovascular or coordination training on volume of the hippocampus. We expected not only cardiovascular training to positively influence hippocampal volume (as shown by Erickson et al., 2011), but also coordination training. This assumption is based on findings in rodents suggesting that motor activity benefits synaptogenesis in activated brain regions (e.g., Black et al., 1990).

## MATERIALS AND METHODS

### PARTICIPANTS

Ninety-two older adults between 62 and 79 years of age were recruited to participate in the *Old Age on the Move* project. Only participants were included who did not report any history of cardiovascular disease, neurological disorder, or motor or cognitive restriction (e.g., metal implants, a score of less than 27 in the Mini Mental Status Examination, MMSE; Folstein et al., 1975). One participant had to be excluded due to cognitive impairment (MMSE <27, cf. O’Bryant et al., 2008 for cut-off level, remaining participants *N* = 91). All subjects participated voluntarily in the study and provided written informed consent to the procedures of the study, which was approved by the ethics committee of the German Psychological Society. The study conformed to the Code of Ethics of the World Medical Association (Declaration of Helsinki).

Participants were tested for normal vision (Freiburg Visual Acuity Test; Bach, 2007) and hearing (simultaneous auditory thresholds at multiple frequencies for both ears; Presentation^®^ software, Neurobehavioral Systems, Albany, Canada; Yund, 2003) and were given a demographics and health questionnaire to determine characteristics of the sample (cf. **Table 1**).

**Table 1:** Demographic information [mean (M) and Standard deviation (SD)] for participants of the cross-sectional sample (*N* = 75) and of the two experimental groups (cardiovascular training, coordination training) and the control group (stretching and relaxation; *N* = 49) at measurement time point *t*1.

	Cross-sectional sample		Cardiovascular training		Coordination training		Control group	
Measure	*M*	SD	*M*	SD	*M*	SD	*M*	SD
Age	68.65	3.54	68.24	2.61	69.63	5.10	68.77	2.56
Proportion females	0.73	0.45	0.71	0.47	0.68	0.48	0.54	0.52
Education	12.15	2.70	13.29	2.69	11.87	3.37	12.15	2.08
BMI	27.27	3.57	27.15	4.08	26.25	2.64	26.18	3.80
Hypertension*	0.41	0.50	0.18	0.39	0.58	0.51	0.23	0.44
ERT	0.24	0.43	0.18	0.39	0.26	0.46	0.23	0.44
Motor fitness	0.00	0.57	0.10	0.61	0.07	0.52	-0.23	0.49
Cardio. fitness	21.59	3.82	22.45	3.05	22.43	3.54	22.43	3.40

Age (average age in years), education (years of education), body mass index (BMI), hypertension (proportion of participants who had been diagnosed with hypertensive disorder), estrogen replacement therapy (ERT, proportion of participants who participated in an estrogen replacement therapy), motor fitness (z-value of overall motor fitness index), cardiovascular (cardio.) fitness (VO_2_ peak ml/kg), *indicates a significant group effect for the intervention groups (p < 0.05).

#### Cross-sectional analysis

For the cross-sectional analysis, we excluded 11 participants from data analysis due to incomplete MRI data or data of quality too low for structural data analysis, and five participants due to incomplete motor data. The remaining 75 participants had a mean age of 68.65 years (SD = 3.54; range 62–79 years; 55 females).

#### Analysis of intervention effects

The 91 participants were randomly assigned to two experimental groups and an active control group with respect to their place of residence and participated in a 12-month intervention study. In addition to the participants excluded from the cross-sectional analysis, we excluded 10 participants from the intervention sample due to incomplete MRI data at measurement time point *t*2 and/or *t*3 or structural MRI data with a quality too low for analysis. From the remaining sample (*N* = 65) we also excluded participants from data analysis (*N* = 16) who were absent for more than 33% of the training sessions. The remaining group consisted of 49 participants between 62 and 79 years (32 women and 17 men, *M* = 68.92, SD = 3.75; cardiovascular training: *N* = 17, 12 women, 5 men; coordination training: *N* = 19, 13 women, 6 men; control group: *N* = 13, 7 women, 6 men; cf. **Table 1** for sample characteristics at *t*1 within each training group). For analysis of selectivity effects see Voelcker-Rehage et al. (2011).

### MOTOR TESTS

#### Fitness assessments

*Motor fitness* at baseline as well as after 6 and 12 months of intervention was assessed by using a heterogeneous battery of eight motor tests representing different domains of motor fitness: flexibility (shoulder flexibility by Rikli and Jones, 1999), action speed (agility by Adrian, 1981; hand tapping by Oja and Tuxworth, 1995; feet tapping by Voelcker-Rehage and Wiertz, 2003), balance (one-leg-stand with eyes open and closed by Ekdahl et al., 1989; backwards beam walk by Kiphard and Schilling, 1974) as well as fine coordination (Purdue Pegboard test by Tiffin and Asher, 1948). An overall index of motor fitness was calculated from these individual performances. For a more detailed description see Voelcker-Rehage et al. (2010). To prove effects of motor coordination training across the 12-month study interval (after 6 and 12 months), we used three tests that characterize motor coordination performance, namely feet tapping (as indicator for action speed), a stick-fall-test (as indicator for reaction speed, Ehrler and Huth, 2000), and one-leg-stand with eyes open (as indicator for balance performance).

*Metabolic fitness* was assessed by calculating an index of grip force (as indicator of muscular strength by Igbokwe, 1992) and peak oxygen consumption (VO_2_ peak, cf. also Voelcker-Rehage et al., 2010). VO_2_ peak as an indicator of cardiovascular fitness was assessed by a spiroergometry (ZAN300, a measurement system of oxygen consumption and for indirect calorimetric assessment). During spiroergometry participants completed a submaximal graded exercise test (a modified Porszasz protocol, Porszasz et al., 2003) on a Lode Valiant motor-driven treadmill with electrocardiography activity monitored by a ten-lead fully digital stress system (Kiss, GE Healthcare, Munich, Germany; cf. Voelcker-Rehage et al., 2010). For training prescriptions (cardiovascular group) we calculated the aerobic (AerTGE) and anaerobic (AnTGE) gas exchange thresholds. The AerTGE of healthy, untrained older people occurred at ~60% VO_2_ peak (Prioux et al., 2000). For data analysis the mean VO_2_ value of the highest complete performance level achieved by the participants, expressed as VO_2_ peak ml/kg, was used. Analyses were also done for cardiovascular fitness only.

#### Interventions

Interventions took place three times per week for 45–60 min. Training interventions were led by an experienced exercise leader and took place in groups of 10–15 participants each.

*Coordination training* focused on the improvement of complex movements for the whole body such as balance, eye–hand coordination, leg–arm coordination as well as spatial orientation and reaction to moving objects/persons. Different fitness equipment such as exercise balls, twist boards, fitness balls, jump ropes, exercise bands, and stability boards were used to offer an individually challenging and diversified program.

*Cardiovascular training* was designed to improve cardiorespiratory fitness via a Nordic Walking program. Training intensity prescriptions were based on heart rate (HR) responses to spiroergometry exercise testing. Participants of the cardiovascular training group trained with their individual HR in an intensity zone at and above AerTGE (but below AnTGE, extensive training, Meyer et al., 2005) as described elsewhere (Voelcker-Rehage et al., 2011).

*Stretching and relaxation training* served as an active control condition to evaluate the potential effects of being involved in a guided group activity for 12 months as well as controlling for retest and social participation effects. A program of relaxation techniques (e.g., imaginary journey, progressive muscle relaxation, and massage), stretching and limbering for the whole body especially designed for older adults was offered to this group. Using different equipment such as fitness balls and rubber tubes provided program variation.

### DESIGN AND MR IMAGE ACQUISITION

At baseline (*t*1), as well as after six (*t*2) and after 12 months (*t*3), each participant completed the fitness assessment in one laboratory session of approximately 2 h. All tasks were administered in a fixed order. On another day but within 1 week, participants underwent the MRI scanning at a 3-Tesla Allegra Scanner. Using a MPRAGE sequence with 1 mm × 1 mm × 1 mm isotropic resolution, a TR of 2300 ms, and 160 slices, T1-weighted anatomical scans were collected. Assessment of the structural MR images was part of a larger MRI testing protocol, the results of which are reported elsewhere (cf. Godde and Voelcker-Rehage, 2010; Voelcker-Rehage et al., 2010, 2011).

### MRI DATA PROCESSING AND ANALYSIS

The T1-weighted MR images were realigned to correct for head tilt, pitch, and rotation passing through the anterior and posterior commissure line (AC-PC alignment) by using Brain Voyager (Brain Innovation B.V., Netherlands). In the next step, hippocampal volumes were measured on coronal sections by manual tracing using Analyze (Analyze Version 10.0, Mayo Clinic, Rochester, MN, USA). Hippocampal volume of the right and left hemisphere as well as intracranial volume (ICV) were defined and traced according to the rules described below (original rules cf. Raz et al., 2003 for the ICV; Raz et al., 2004b for the hippocampus). Then, volumes were computed by multiplying the sum of the areas within each slice by slice thickness. Finally, individual (i) raw volumes were adjusted using the following formula: Volume_adj_i_ = volume_raw_i_ - b (ICV_i_ - ICV_samplemean_; Raz et al., 2003) to correct for inter-individual ICV differences, where b is the regression slope of volume_raw_i_ on ICV. Reliability of volume determination was secured by calculating the intraclass correlation coefficient ICC (2; for more information on the tracing reliability process see Raz et al., 2004a). All ICC(2) values were above the critical level of.90.

Intracranial volume was estimated by tracing its volume on every 10th image slice (approx. 16 slices) between the first slice following the orbits and the last slice on which brain tissue was visible. This method has shown to save a lot of time (in comparison with tracing every slice) by only having negligible reductions in reliability (Eritaia et al., 2000). Tracing started at the right side of the head proceeding clockwise while following the inner table of the skull until the homologous mark on the left side of the head was reached. At this point, tracing was stopped and automatically a straight line back to the origin was drawn by the program (cf. Raz et al., 2003).

**FIGURE 1:**
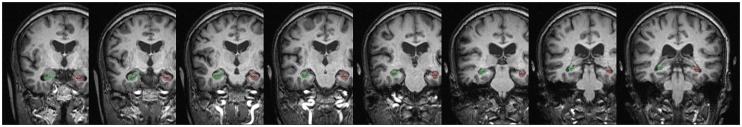
**Example images and representative analyses of hippocampal tracing rules from anterior to posterior brain sections**. Marked structure on the right-hand side of the pictures represents hippocampal formation of left hemisphere, marked structure on the left-hand side on the pictures represents hippocampal formation of right hemisphere.

Hippocampal volume was estimated on every slice from the first slice where the mammillary bodies appear most bulbous. The posterior boundary was determined by looking for the slice that cuts through the fornices as they rise after leaving the fimbria of the hippocampus and then using the next slice as the last one (cf. Raz et al., 2004b). The volume of the hippocampus was estimated from 22 to 32 coronal sections. Further demarcation lines were identical as described by Raz et al. (2004a; cf. **Figure 1** for example images).

### STATISTICAL ANALYSES

#### Cross-sectional analysis (t1)

We conducted two linear regression analyses to investigate the relationship between motor fitness, metabolic fitness, and the interaction between motor and metabolic fitness (independent variables) as well as motor fitness, cardiovascular fitness and their interaction term (independent variables) on hippocampal volume (dependent variable), respectively. Hippocampal volume did not correlate with age, sex, years of formal education, subjective health, and estrogen replacement therapy. However, as motor fitness correlated with age and sex, we included those variables in the regression analyses.

#### Analysis of intervention effects

To investigate the effects of the intervention programs on cardiovascular fitness, a 3 × 3 mixed factor ANOVA with repeated measures was computed with SESSION (*t*1, *t*2, *t*3) as the within-subject variable and GROUP (cardiovascular training, coordination training, control group) as the between-subject variable. Intervention effects on motor fitness were analyzed by a 3 (SESSION: *t*1, *t*2, *t*3) × 3 (motor fitness TASK: action speed, reaction speed, one-leg-stand with eyes open) × 3 (GROUP: cardiovascular training, coordination training, control group) repeated-measures ANOVA. Furthermore, a 3 × 3 mixed factors ANOVA with repeated measures was computed with SESSION (*t*1, *t*2, *t*3) as the within-subject variable and GROUP (cardiovascular training, coordination training, control group) as the between-subject variable to investigate the effects of the intervention programs on total hippocampal volume (sum of right and left HC volume). We further analyzed hippocampal volume of the left and right hemisphere separately to test for differences of intervention effects on both hemispheres. Greenhouse Geisser adjustment is reported in case the sphericity assumption was violated. To determine effects within the exercise groups, analyses were computed separately for each exercise group using estimated marginal means collapsed over the between and within-subject variables. *Post hoc* pairwise comparisons with Bonferroni adjustment were conducted to determine pre-to-post-test changes within and between the experimental and control groups. Effect sizes were calculated for significant results by partial eta-square (η^2^), expressing the amount of variance explained in the dependent variables by the respective effect. Experimental and control groups were statistically similar to one another on measures of age, sex, years of formal education, BMI, hypertension, and estrogene replacement therapy (for women) as well as motor and cardiovascular fitness (cf. **Table 1**), and did not reveal a noticeable selectivity effect (for IQ, health, activity index and positive affect cf. Voelcker-Rehage et al., 2011). Thus, we abstained from including covariates.

Bringing together changes in fitness domains and hippocampal volume after the intervention, we calculated three linear regression analyses with hippocampal volume at *t*3 (sum of both hemispheres, left HC, right HC) as dependent variable and changes in motor tests from *t*1 to *t*3 (action speed, balance, and cardiovascular fitness) as independent variables. Reaction speed did not show significant changes and was therefore not included in the regression analysis. Hippocampal volume and change scores (*t*3 minus *t*1) did not correlate with possible covariates, thus, we abstained from including other covariates in the analyses. For all analyses, the significance level was set to α = 0.05.

## RESULTS

### CROSS-SECTIONAL DATA: ASSOCIATION OF FITNESS WITH VOLUME OF THE HIPPOCAMPUS

**FIGURE 2:**
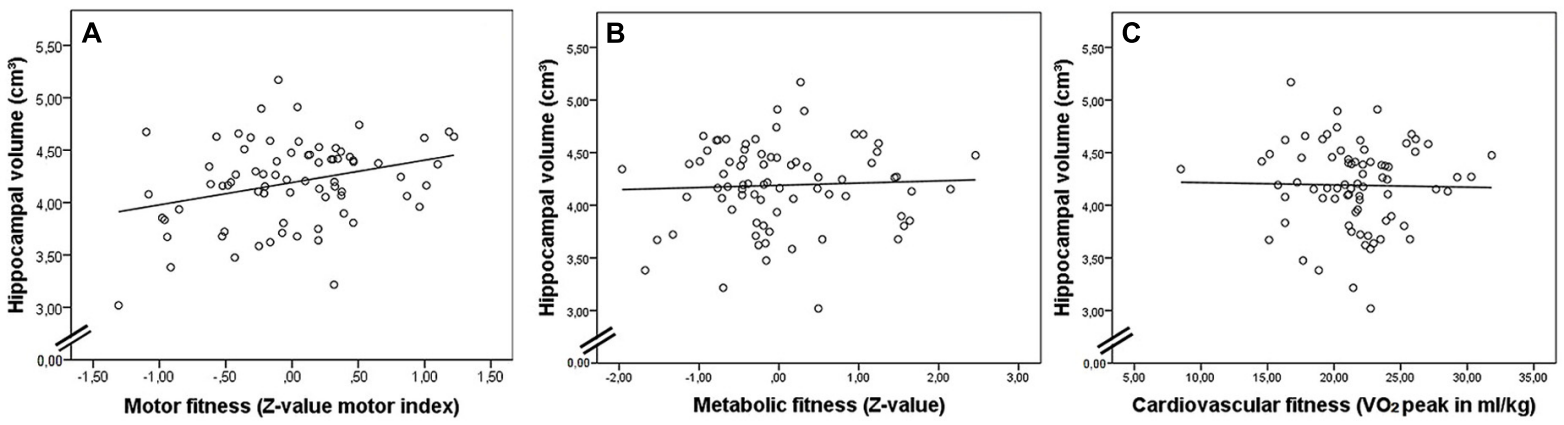
**Scatterplots showing sum of hippocampal volume of both hemispheres (in cm^3^) in relationship with **(A)** motor fitness (significant), **(B)** metabolic fitness, and **(C)** cardiovascular fitness at *t*1**.

Linear regression analysis revealed that fitness dimensions and their interaction as well as age and sex together explained 10.9% of variance in hippocampal volume (see **Table 2**). Motor fitness uniquely explained 7% of variance in hippocampal volume, whereas the other variables did not further explain variance in hippocampal volume significantly (shared variance 1.5%; cf. additionally **Figure 2** for a scatterplot of hippocampal volume against motor fitness (**Figure 2A**) and metabolic fitness (**Figure 2B**). The second regression analysis with cardiovascular fitness instead of metabolic fitness revealed comparable results explaining 13.1% of total variance in hippocampal volume (see **Table 3** and **Figure 2C** for a scatterplot of hippocampal volume against cardiovascular fitness). Again, motor fitness uniquely explained 9% of variance in hippocampal volume and none of the other variables did significantly explain additional variance.

**Table 2:** Regression statistics for hippocampal (HC) volume (sum HC volume: sum of left and right volume, left HC volume, right HC volume) as dependent variable and age, sex, motor fitness (MF), metabolic fitness (MBF), and their interaction term as independent variables.

	Sum HC volume			Left HC volume			Right HC volume		
Measure	*B*	SE B	β	*B*	SE B	β	*B*	SE B	β
Constant	4889.41	945.21		2653.36	517.30		2235.63	519.82	
Age	-12.88	14.16	-0.11	-9.53	7.75	-0.15	-3.35	7.79	-0.06
Sex	147.64	182.86	0.16	92.70	100.08	0.19	55.09	100.57	0.11
MF	211.68	91.25	0.30*	105.29	49.94	0.27*	106.42	50.18	0.28*
MBF	-42.60	88.21	-0.10	-22.63	48.28	-0.09	-19.93	48.51	-0.08
MF × MBF	-33.34	97.27	-0.04	-33.14	53.24	-0.07	-1.18	53.50	-0.00

Sum HC volume: R^2^ = 0.11 (p = 0.15), Left HC volume: R^2^ = 0.12 (p = 0.13), Right HC volume: R^2^ = 0.08 (p = 0.34), *p < 0.05.

**Table 3:** Regression statistics for hippocampal (HC) volume (sum HC volume: sum of left and right volume, left HC volume, right HC volume) as dependent variable and age, sex, motor fitness (MF), cardiovascular fitness (CF), and their interaction term as independent variables.

	Sum HC volume			Left HC volume			Right HC volume		
Measure	*B*	SE B	β	*B*	SE B	β	*B*	SE B	β
Constant	4690.50	955.80		2520.30	522.60		2169.76	528.34	
Age	-10.69	13.98	-0.09	-8.19	7.65	-0.13	-2.50	7.73	-0.04
Sex	178.60	125.52	0.20	121.75	68.63	0.25	57.06	69.38	0.12
MF	250.41	93.49	0.35*	132.14	51.12	0.34*	118.31	51.68	0.31*
CF	-55.43	56.89	-0.14	31.62	50.68	-0.08	-18.17	31.45	-0.08
MF × CF	69.97	92.68	0.09	-37.26	31.10	-0.17	38.34	51.23	-0.09

Sum HC volume: R^2^ = 0.13 (p = 0.08), Left HC volume: R^2^ = 0.14 (p = 0.06), Right HC volume: R^2^ = 0.09 (p = 0.25), *p < 0.05.

Results separately for both hemispheres are similar as for total hippocampal volume (cf. **Tables 2** and **3**; for the left hippocampus, all variables accounted for 11.5% of variance in volume; for the right hemisphere the explained variance was 7.7%. Motor fitness uniquely explained 5.7% of the variance in left hippocampal volume and 6% in right hemisphere. Neither metabolic fitness, cardiovascular fitness or the respective interaction terms with motor fitness nor the control variables age and sex did explain additional variance.

### EFFECTS OF CARDIOVASCULAR AND COORDINATION TRAINING ON FITNESS

**FIGURE 3:**
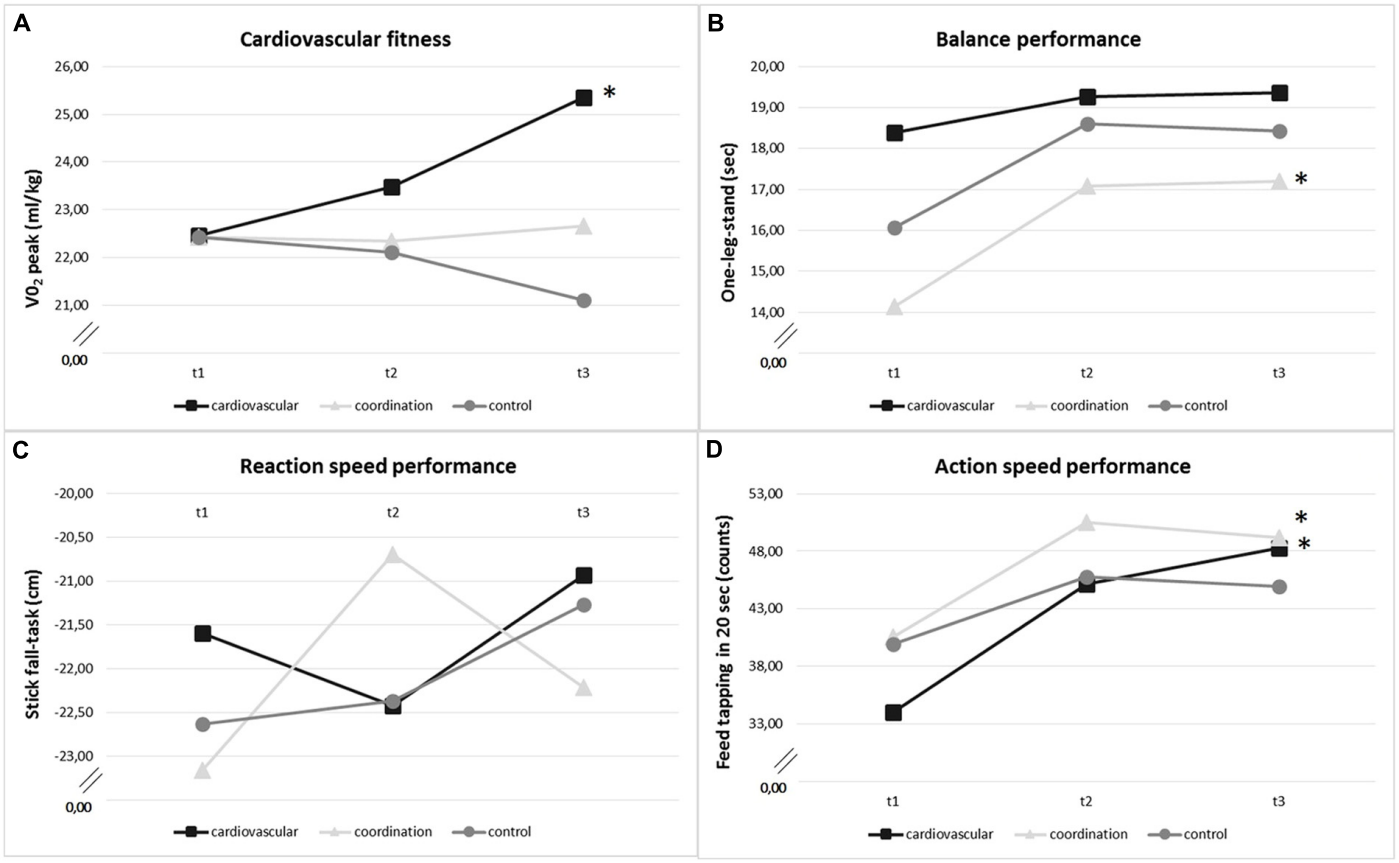
**Change of (A) cardiovascular fitness (VO_2_ peak in ml/kg), **(B)** balance performance (one-leg-stand in seconds, max 20 sec.), **(C)** reaction speed performance (stick-fall-task in cm), and **(D)** action speed performance (feet tapping in 20 sec.) within training groups (cardiovascular training, coordination training, control group) over the intervention period (*t*1: baseline, *t*2: after 6 months, *t*3: after 12 months).** *Significant change (*p* < 0.05).

To compare efficiency of the specific training programs we analyzed training effects on cardiovascular and motor fitness, respectively, for all three study groups (cf. **Figure 3A** and **Table 4**). Overall, for cardiovascular fitness we found a significant SESSION × GROUP interaction [*F*(3.55,81.54) = 3.68, *p* = 0.01, η^2^ = 0.14], but no SESSION [*F*(1.77,81.54) = 0.93, *p* = 0.40, η^2^ = 0.02] or GROUP effect [*F*(2,46) = 1.15, *p* = 0.33, η^2^ = 0.05]. Separate analyses for the three groups revealed that cardiovascular fitness in the cardiovascular training group significantly increased by 13.65% across the 12-month intervention [*F*(2,45) = 5.58, *p* = 0.01, η^2^ = 0.20] with *post hoc* pairwise comparisons showing significant improvements only from *t*2 to *t*3 (*p* < 0.01), but not from *t*1 to *t*2. No significant changes could be observed in the coordination training group [1.98% increase; *F*(2,45) = 0.16, *p* = 0.88, η^2^ = 0.01] and the control group [5.65% decrease; *F*(2,45) = 0.98, *p* = 0.38, η^2^ = 0.04].

**Table 4:** Mean (M) and Standard deviation (SD) for motor fitness (*z*-value of overall motor fitness index) and cardiovascular (cardio.) fitness for all three measurement time points (*t*1, *t*2, *t*3) and for the two experimental groups (cardiovascular training, coordination training) and the control group (stretching and relaxation).

		Motor fitness		Cardio fitness	
Group	Session	*M*	SD	*M*	SD
Cardiovascular training	*t*1	0.10	0.61	22.45	3.05
	*t*2	0.33	0.65	23.48	4.10
	*t*3	0.37	0.65	25.35	3.55
Coordination training	*t*1	0.07	0.52	22.43	3.54
	*t*2	0.26	0.58	22.34	4.17
	*t*3	0.29	0.50	22.66	5.15
Control group	*t*1	-0.23	0.49	22.43	3.40
	*t*2	0.00	0.53	22.11	3.59
	*t*3	0.00	0.52	21.12	4.36


For the overall change in motor tasks, we found a main effect of SESSION [*F*(1.70,78.25) = 31.38, *p* < 0.001, η^2^ = 0.41] and TASK [*F*(1.37,63.16) = 1538.42, *p* < 0.001, η^2^ = 0.97] and a significant SESSION × TASK interaction [*F*(2.40,110.48) = 10.47, *p* < 0.001, η^2^ = 0.19]. There was no GROUP effect [*F*(2,46) = 0.07, *p* = 0.93, η^2^ = 0.00] and no GROUP × SESSION interaction [*F*(3.40,78.25) = 1.43, *p* = 0.24, η^2^ = 0.06], no GROUP × TASK interaction [*F*(2.75,63.16) = 1.78, *p* = 0.16, η^2^ = 0.07], and no GROUP × SESSION × TASK interaction [*F*(4.80,110.48) = 1.31, *p* = 0.24, η^2^ = 0.05]. Calculating separate analyses for the study groups showed that after the 12-month intervention all study groups significantly improved their performance in the three selected motor fitness tasks [cardiovascular group: *F*(2,45) = 11. 22, *p* < 0.001, η^2^ = 0.33; coordination group: *F*[2,45] = 18.44, *p* < 0.001, η^2^ = 0.45; control group: *F*(2,45) = 4.03, *p* = 0.03, η^2^ = 0.15]. Analyses separately for the three tasks action speed, reaction speed and balance revealed a different development for motor fitness indicators within the three groups (cf. **Figures 3B–D**). The cardiovascular training group improved their action speed performance by 42% [*F*(2,45) = 12.62, *p* < 0.001, η^2^ = 0.36; with significant improvements from *t*1 to *t*2 (*p* < 0.001), but not *t*2 to *t*3]. Similarly, the coordination training group revealed an increase in action speed performance of 21.1% [*F*(2,45) = 8.35, *p* < 0.001, η^2^ = 0.27; with significant improvements from *t*1 to *t*2 (*p* < 0.001), but not from *t*2 to *t*3] and in addition an 21.6 % improvement of performance in one-leg-stand with eyes open [*F*(2,45) = 5.25, *p* = 0.01, η^2^ = 0.19; with significant improvements from *t*1 to *t*2 (*p* < 0.01), but not from *t*2 to *t*3]. Changes in reaction speed did not reach significance level (cardiovascular group: 3.6% improvement [*F*(2,45) = 1.31, *p* = 0.28, η^2^ = 0.06]; coordination group: 8.4% improvement [*F*(2,45) = 0.88, *p* = 0.42, η^2^ = 0.59)]. The control group did not show any significant changes across the 12-month intervention period in these three tasks.

### EFFECTS OF CARDIOVASCULAR AND COORDINATION TRAINING ON VOLUME OF THE HIPPOCAMPUS

#### Hippocampal volume

**FIGURE 4:**
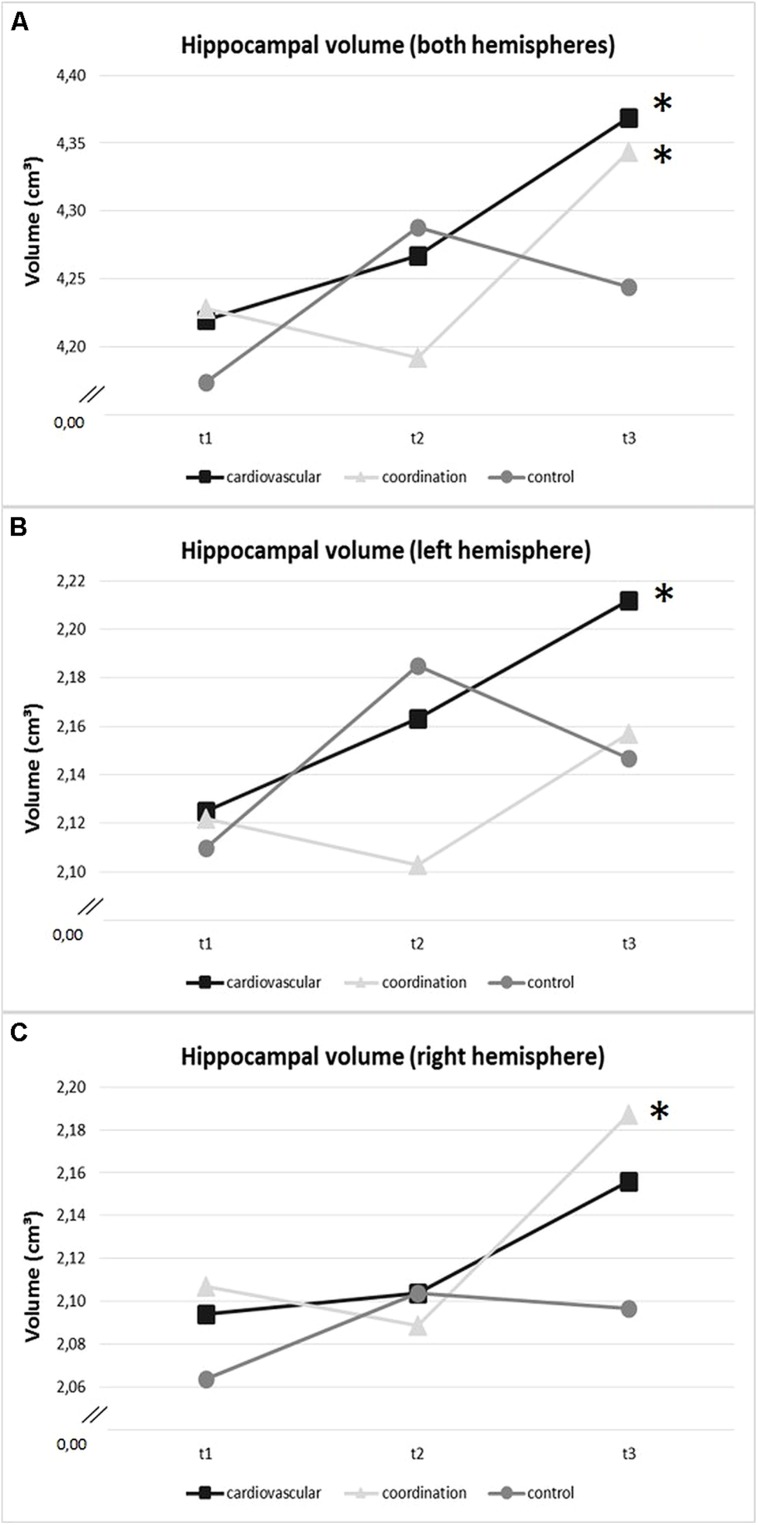
**Change in volume of (A) hippocampal volume of both hemispheres, **(B)** left hippocampus, and (C) right hippocampus (in cm^3^) within training groups (cardiovascular training, coordination training, control group) over the intervention period (*t*1: baseline, *t*2: after 6 months, *t*3: after 12 months).** *Significant change (*p* < 0.05).

Regarding the intervention effects on the hippocampal volume (sum score of volume of right and left hemisphere), the repeated measures ANOVA with three measurement time points (*t*1, *t*2, *t*3) revealed a main effect for SESSION, but no GROUP effect and no GROUP × SESSION interaction (cf. **Table 5**). Analyses separated by exercise groups revealed that in the cardiovascular group hippocampal volume significantly increased by 3.60% [*F*(2,45) = 4.76, *p* = 0.01, η^2^ = 0.17] across the 12-month exercise intervention (cf. also **Table 6**). *Post hoc* pairwise comparisons revealed only a significant overall increase, but no significant changes in hippocampal volume from *t*1 to *t*2 or from *t*2 to *t*3 (cf. **Figure 4A** and **Table 6**). Further, there was a significant increase of hippocampal volume (2.84%) after the coordination training [*F*(2,45) = 4.73, *p* = 0.01, η^2^ = 0.17; with significant improvements from *t*2 to *t*3 (*p* < 0.05), but not from *t*1 to *t*2; cf. **Figure 4A** and **Table 6**]. Volume increase across the 12 months in the control group (1.67%) was not significant [*F*(2,45) = 1.63, *p* = 0.21, η^2^ = 0.07; cf. **Figure 4A** and **Table 6**].

**Table 5:** Statistics for repeated measure ANOVA with main effects SESSION (*t*1, *t*2, *t*3) and GROUP (cardiovascular training, coordination training, control group) and interaction SESSION × GROUP for hippocampal (HC) volume (sum HC: sum of left and right volume, left HC volume, right HC volume).

	SESSION				GROUP				SESSION × GROUP			
Measure	*F*	df	*p*	η^2^	*F*	df	*p*	η^2^	*F*	df	*p*	η^2^
Sum HC	5.78	2, 92	<0.001***	0.11	0.05	2, 46	0.95	0.00	1.71	4, 92	0.16	0.07
Left HC	3.72	2, 92	0.03*	0.08	0.14	2, 46	0.87	0.01	1.61	4, 92	0.18	0.07
Right HC	6.18	2, 92	<0.001***	0.12	0.12	2, 46	0.89	0.01	1.44	4, 92	0.23	0.06


*p < 0.05; ***p < 0.001.

**Table 6:** Mean (M) and Standard deviation (SD) for hippocampal volume in cm^**3**^ collapsed over both hemispheres (sum HC: sum of left and right volume) and separately for left (left HC volume) and right hemisphere (right HC volume) for all three measurement time points (*t*1, *t*2, *t*3).

		Sum HC volume		Left HC		Right HC	
Group	Session	*M*	SD	*M*	SD	*M*	SD
Cardiovascular training	*t*1	4.22	0.41	2.13	0.22	2.09	0.21
	*t*2	4.27	0.44	2.16	0.23	2.10	0.24
	*t*3	4.37	0.45	2.21	0.24	2.16	0.25
Coordination training	*t*1	4.23	0.40	2.12	0.22	2.11	0.22
	*t*2	4.19	0.38	2.10	0.21	2.09	0.22
	*t*3	4.34	0.44	2.16	0.24	2.19	0.24
Control group	*t*1	4.17	0.48	2.11	0.27	2.06	0.27
	*t*2	4.29	0.51	2.19	0.27	2.10	0.29
	*t*3	4.24	0.53	2.15	0.30	2.10	0.27

#### Left and right hippocampal hemisphere separately

Results for the left and right hemisphere confirmed results for the total hippocampal volume with a significant main effect for SESSION, but neither a GROUP effect nor a GROUP × SESSION interaction (cf. **Table 5**). Analyses separated by exercise groups revealed that only the cardiovascular group significantly increased hippocampal volume of the left hemisphere by 4.22% [*F*(2,45) = 5.10, *p* = 0.01, η^2^ = 0.19] across the 12 months exercise intervention (only significant overall change, but no changes from *t*1 to *t*2 or from *t*2 to *t*3; cf. **Figure 4B**). Analyses for the coordination and control groups revealed only non-significant increases of 1.78% and 1.65%, respectively. Interestingly, the coordination group significantly increased hippocampal volume of the right hemisphere by 3.91% [*F*(2,45) = 6.03, *p* = 0.01, η^2^ = 0.21] across the 12-month exercise intervention [with a significant change from *t*2 to *t*3 (*p* < 0.01), but not from *t*1 to *t*2, cf. **Figure 4C**]. Analyses for the cardiovascular and control groups revealed only non-significant increases of 2.98% and 1.68% volume of the right hippocampus, respectively.

### RELATIONSHIP BETWEEN CHANGES IN FITNESS AND HIPPOCAMPAL VOLUME

Using a linear regression analysis with total hippocampal volume at *t*3 as dependent variable, only change in cardiovascular fitness (VO_2_ peak ml/kg) significantly explained variance (9.3%, *p* = 0.04) in hippocampal volume (cf. **Table 7**). When analyzing both hemispheres separately, again, only VO_2_ peak significantly explained 12.1% (*p* = 0.02) of variance in volume of the left hippocampus (cf. **Table 7**) – the side of the hippocampus that revealed a volume increase in the cardiovascular training group. No such effect was found for the right hemisphere.

**Table 7:** Regression statistics for hippocampal volume at *t*3 (sum HC volume: sum of left and right volume, left HC volume, right HC volume) as dependent variable and changes in motor fitness domains that showed significant changes (Δ Balance, Action speed) and changes in cardiovascular fitness (Δ VO_**2**_ peak) from *t*1 to *t*3 as independent variables.

	Sum HC volume			Left HC volume			Right HC volume		
Measure	*B*	SE B	β	*B*	SE B	β	*B*	SE B	β
Constant	42.25	32.04		14.85	17.34		27.35	17.56	
Δ Balance (sec.)	6.50	7.53	0.12	2.07	4.07	0.07	4.43	4.13	0.15
Δ Action speed (counts)	-0.26	2.65	0.01	-0.57	1.44	-0.06	0.32	1.45	0.03
Δ VO_2_ peak ml/kg	18.08	8.30	0.31*	11.25	4.49	0.35*	6.83	4.55	0.22

Sum HC volume: R^2^ = 0.12 (p = 0.13). Left HC volume: R^2^ = 0.13 (p = 0.10). Right HC volume: R^2^ = 0.08 (p = 0.29); *p < 0.05.

## DISCUSSION

We investigated whether metabolic/cardiovascular and motor fitness are associated with hippocampal volume in healthy older adults and whether a 12-month cardiovascular or coordination training induces changes in hippocampal volume in these participants. Cross-sectional data indicate that only motor fitness (not cardiovascular fitness or metabolic fitness) is associated with hippocampal volume. However, after 12 months of training, both types of exercise, cardiovascular and coordination training, positively but differently, influence volume of the hippocampus.

### CROSS-SECTIONAL ANALYSES

In line with our hypothesis for the cross-sectional analyses, motor fitness was significantly associated with hippocampal volume accounting for 7% of explained variance. To our knowledge, this is the first study that described a relationship between hippocampal volume and motor fitness, i.e., the component of fitness that requires perceptual and higher-level cognitive processes. Herewith we are adding significantly to previous functional brain data revealing a positive association between motor fitness and functional brain activation in frontal and parietal areas accompanied by better performance in perceptual speed and executive functions (Voelcker-Rehage et al., 2010).

We could not demonstrate a relationship between hippocampal volume and metabolic fitness (comprising cardiovascular fitness and grip strength) or cardiovascular fitness alone. This is in line with cross-sectional studies by Honea et al. (2009), Flöel et al. (2010), and Smith et al. (2011) who also did not find correlations between volume of the medial temporal lobe area, or more specifically the hippocampus, and cardiovascular fitness in different samples of healthy older adults (50+ years of age). However, a relationship between those factors was assumed by Erickson et al. (2009) as well as by Szabo et al. (2011) who both demonstrated a positive association between higher cardiovascular fitness and larger hippocampal volume in samples of healthy American older adults. Whereas our sample did not differ from theirs in age and presumably the level of cardiovascular fitness, differences in data analysis might account for diverging results: it has been shown that using (semi-)automated computer-programs (e.g., VBM, FIRST, cf. Erickson et al., 2009; Szabo et al., 2011) might reveal different results regarding brain volume than tracing predetermined brain regions manually (cf. Kennedy et al., 2009 for a comparison of different analyzing methods). Interestingly, Honea et al. (2009) confirmed a correlation between medial temporal lobe and cardiovascular fitness in patients suffering from early stages of Alzheimer‘s disease (Honea et al., 2009). Overall, although associations between hippocampal volume and cardiovascular fitness might be plausible, results are not such consistent and might depend on reasons mentioned above or on moderating variables still not identified.

### INTERVENTION STUDY

Data of the 12-month intervention study revealed a positive effect on total hippocampal volume (sum scores of right and left hemisphere) for both types of exercise, cardiovascular and coordination training. Interestingly, our findings for cardiovascular training mirror the results of Erickson et al. (2011) who also found an increase in hippocampal volume after 12 months of cardiovascular training, although cross-sectional results were different (see above). Possibly, training effects in a controlled trial are more reliable and stronger than cross-sectional associations of hippocampal volume and cardiovascular fitness that are influenced to a higher degree by sample characteristics and further uncontrolled factors.

Whereas Erickson et al. (2011) revealed an increase in left and right hippocampal volume by 2.12% and 1.97%, respectively, we found an even higher (twice as high) volume increase in the left hippocampus of 4.22% and an increase of 2.98% for the right hippocampus. The increase in the right hippocampus was, however, not statistically significant in our cardiovascular training group, probably due to the high variability within our sample. The higher level of volume increase, particularly for the left hippocampus, in our study might be explained by the magnitude of change in VO_2_ peak performance. In our sample, participants of the cardiovascular training group improved by 13.65%, whereas Erickson et al. (2011) found increases of cardiovascular fitness only by 7.78%. As Erickson et al. (2011) and our sample support the assumption that there might be an association between change in VO_2_ peak and hippocampal volume at post-test, one might suggest that there is a linear relationship between both variables.

### EFFECTS OF COORDINATION TRAINING

In addition, for the first time, we showed that not only changes in cardiovascular fitness seem to be associated with hippocampal volume in older adults, but also a 12-month coordination training led to changes in total hippocampal volume. In contrast to cardiovascular training, these changes were more pronounced in the right hippocampus (right: 3.91%; left: 1.78% non-significant change). Particularly the right hippocampus is known to be involved in spatial memory processes (Smith and Milner, 1981; Nunn et al., 1999; Burgess et al., 2002). As coordinative exercises to a high degree rely on and practice spatial orientation, this might be one explanation for our finding.

Studies in young adults have shown that short-term motor learning (e.g., performing a balance task, learning to write with the left non-dominant hand, etc.) seems to induce very rapid volume changes (increases and decreases within two to six training sessions) in training-related brain regions (Taubert et al., 2010; Gryga et al., 2012; Hamzei et al., 2012). We could not reveal changes in hippocampal volume after 6 months of training, but found a significant increase in volume after the second half of the intervention. These results might indicate that rapid volume changes might fluctuate in the beginning of training (Taubert et al., 2010; Gryga et al., 2012) and, since patterns of plastic changes might differ in regard to training duration (cf. Hamzei et al., 2012), are more stabilized after the continued training of complex motor tasks. In contrast to the cardiovascular training, we could not identify changes in specific motor tasks to cause changes in hippocampal volume. One reason might be that the changes in the motor system due to coordination training were not adequately measured by the selected motor tests.

In addition, in animal research, so far, few studies have compared effects of cardiovascular training and motor-demanding activities (corresponding to coordination training in humans, cf. Voelcker-Rehage and Niemann, 2013 for a further discussion). Greenough and colleagues (Black et al., 1990; Isaacs et al., 1992; Anderson et al., 1994) provided evidence that cardiovascular training increases capillary density in rodents, while acrobatic training in rodents induces the generation of new synapses and glia cells as well as dendritic hypertrophy (cf. Kleim et al., 1996, 1997, 1998; Seo et al., 2010). The motor-based training initiated blood vessel growth only to maintain the diffusion distance (Isaacs et al., 1992). Diverging underlying mechanisms might also explain the different time courses of volume change that we observed after the two types of exercise training. While the cardiovascular training group showed almost linear increase in hippocampal volume, hippocampal volume of the coordination training group increased from month 6 to 12 of the intervention only (cf. **Figure 4**). A recent review (Thomas et al., 2012) provided time courses for the different components of brain plasticity, i.e., angiogenesis and capillary density, neurogenesis, astrocyte volume, and neuropil volume. Following them, angiogenesis as well as neurogenesis show a rapid time course. For example, in mice capillary density is already enhanced after three days of increased aerobic activity, but also returns to baseline within 24 h of sedentary behavior (Van der Borght et al., 2009). Similarly, in rats astrocyte changes occur rapidly but return to baseline volume as soon as activity is stopped (Kleim et al., 2007). In contrast, again in rats changes in the neuropil are more stable and might last for several weeks (Kleim et al., 1997, 4 weeks after removed from enriched environment; see also a review by Markham and Greenough, 2004). Although to date, it is not terminally resolved whether the time course in brain plasticity is transferable from studies in rodents to humans, these findings from the animal literature let us suggest that also different underlying mechanisms might be responsible for the volume changes accompanied by different time courses in our sample of healthy older adults.

In addition, animal studies focusing on neurogenesis in the hippocampus support our diverging findings for cardiovascular and coordination training on hippocampal volume. They indicate that different types of activity stimulate the generation of new neurons in different ways (van Praag et al., 1999). In rodents, both types go in line with an enhancement of cell numbers: cardiovascular activity induces the proliferation of newborn cells in the hippocampus to a large extent. In contrast, an “enriched environment” condition consisting of a complex combination of physical, motor and social stimulation also promoted the proliferation of new born cells (approx. 50% of cardiovascular training effects) leading, in the long run, to a higher degree of integration and survival of the neurons (van Praag et al., 1999; Curlik et al., 2013). This finding could also add to our results in humans after 6 months of training, where we found changes in the cardiovascular group but not the coordination group and might explain why we found comparable volume increases after 12 months of intervention for both types of training. However, as we do not have follow up data after cessation of the exercise training, we, unfortunately, do not have any information about how long exercise changes last.

## CONCLUSION

We were able to show that strengthening motor fitness and engaging in regular exercise (cardiovascular or coordination training) is beneficial for hippocampal volume in older adults. We herewith add to the exercise and cognition literature that mainly focuses on cardiovascular fitness another promising fitness component that is worth to be further investigated, namely motor fitness and coordination training. Although first studies are able to show, that cardiovascular activity-related increases in hippocampal volume in older adults go in line with benefits in spatial memory performance (cf. Erickson et al., 2009, 2011), further research is needed to clarify whether slowing down the shrinkage of the hippocampus volume might also diminish decline in hippocampal-dependent cognitive functioning in healthy older adults as well as people suffering from mild cognitive impairment or early stages of Alzheimer’s disease.

## Conflict of Interest Statement

The authors declare that the research was conducted in the absence of any commercial or financial relationships that could be construed as a potential conflict of interest.
